# Co-infection with *Bartonella henselae* and *Sarcocystis sp.* in a 6-year-old male neutered domestic longhair cat with progressive multifocal neurological signs

**DOI:** 10.1080/01652176.2019.1697012

**Published:** 2019-12-10

**Authors:** Aude Castel, Natasha J. Olby, Edward B. Breitschwerdt, Brittany Thomas, Ricardo G. Maggi, G. Diane Shelton

**Affiliations:** a Department of Clinical Sciences, College of Veterinary Medicine, North Carolina State University, Raleigh, NC, USA;; b The Comparative Medicine Institute, College of Veterinary Medicine, North Carolina State University, Raleigh, NC, USA;; c The Intracellular Pathogens Research Laboratory, College of Veterinary Medicine, North Carolina State University, Raleigh, NC, USA;; d The Department of Pathology, School of Medicine, University of California, Irvine, CA, USA

**Keywords:** cat, feline, *Sarcocystis caninum*, *Sarcocystis arctica*, *Sarcocystis felis: Bartonella henselae*, feline infectious myositis, dysphagia, chorioretinitis, spastic pupil syndrome

A 6-year-old male neutered domestic longhair cat presented to our Neurology Service for changing anisocoria and progressive gait abnormalities. The cat was rescued at about 5 months of age and had been strictly indoor since. A year previously, the cat was evaluated by our Internal Medicine Service for an acute onset of vomiting, dysphagia, dysphonia, inspiratory stridor and suspected bilateral laryngeal paralysis. Physical examination revealed mild anisocoria (right eye (OD) miosis), dysphonia and right thoracic limb (RTL) lameness. Eosinophilia, hyperglobulinemia and hypocholesterolemia were the only complete blood count (CBC) and serum biochemical abnormalities (Supplemental documents). Serum protein electrophoresis confirmed a polyclonal gammopathy. Diagnostic testing, including FeLV/FIV ELISA, acetylcholine receptor antibody titer, abdominal ultrasound, splenic aspirates cytopathology and a swallowing study, did not identify a cause for the clinical signs. The cat improved without treatment within a week. Historically, anisocoria (miosis OD) was reported since a kitten. However, 3 weeks before presentation to the Neurology Service, the cat developed mydriasis OD and left eye (OS) miosis (Figure 1), reflecting a reversal in the “historical” anisocoria. One week later, the cat developed Horner’s syndrome OD, pelvic limb ataxia and knuckling on its left thoracic limb (LTL), prompting referral.

**Figure 1.**
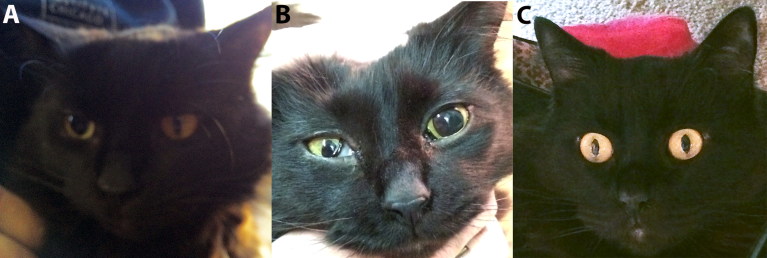
Pupillary changes consistent with spastic pupil syndrome in a 6-year-old male neutered cat. A. Picture of the cat about 48 h prior to presentation (early February 2015) showing anisocoria with the left pupil being smaller than the right pupil. B. The cat in late February 2015, showing a change in the anisocoria with the right pupil being smaller than the left pupil. According to the owner, this was the pattern of anisocoria that the cat had since a kitten, i.e. for six years. Also note the nictitating membrane protrusion consistent with Horner’s syndrome. C. This picture shows the cat in June 2015, after complete resolution of all clinical and neuromuscular signs including the historical anisocoria.

Physical examination abnormalities included miosis OD with prolapsed nictitating membrane and mild muscle atrophy of the LTL. Examination in the dark did not abolish the anisocoria (persistent miosis OD). Fundic examination revealed bilateral hyperreflective tapetal lesions secondary to chorioretinitis (Supplemental documents). Neurologic examination findings included pelvic limb ataxia, a plantigrade stance and intermittent knuckling with a palmigrade stance on the LTL. Proprioceptive deficits and reduced withdrawal reflex on the LTL were noted. Spinal reflexes and cranial nerve function were normal. These findings suggested a multifocal disease involving the left cervical intumescence, the sympathetic innervation of the right eye and possible pelvic limb neuromuscular involvement. Eosinophilia, hyperglobulinemia and hypocholesterolemia were persistent (Supplemental documents).

When rechecked 3 days later, the cat’s anisocoria had changed side, consistent with spastic pupil syndrome. Magnetic resonance imaging (1.5 Tesla, Siemens Medical Solutions, Malvern, PA, USA) of the cervical spine revealed diffuse contrast enhancement of the supraspinatus, subscapularis and triceps musculature bilaterally, worse on the left, with no evidence of spinal cord involvement (Figure 2). Cisternal cerebrospinal fluid (CSF) was normal but there was albuminocytological dissociation of lumbar CSF (nucleated cell count (NCC) 4/µL, reference value <5; protein concentration: 1.24 g/L, reference value <0.4) with a cytological increase in the proportion of non-degenerate neutrophils (80% neutrophils, 9% lymphocytes and 11% macrophages). CSF and blood Toxoplasma, Cryptococcus and Coronavirus titers and blood FelV/FIV ELISA results were negative. *Bartonella henselae* DNA was amplified and sequenced from a Bartonella Alpha Proteobacteria Growth Medium (BAPGM) enrichment blood culture by Galaxy Diagnostics Inc. Research Triangle Park, NC, USA. The 16S-23S intergenic spacer DNA sequence was 100% similar (377 of 377 bp) to *Bartonella henselae* Houston 1 (Gen Bank accession number CP020742). Conversely, BAPGM CSF culture as well as a quantitative PCR on blood and CSF were PCR-negative. The cat was discharged with instructions to administer oral clindamycin at 15 mg/kg BW every 12 h for potential toxoplasmosis, but the medication was discontinued after a week based upon negative *Toxoplasma gondii* titers (by immunofluorescent assay (IFA)) for immunoglobulin M (IgM) and immunoglobulin G (IgG)).

**Figure 2.**
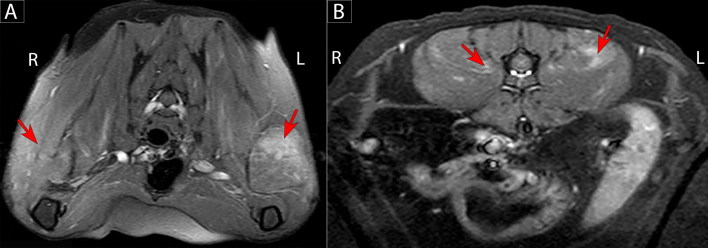
Transverse T1-weighed post-contrast with fat saturation magnetic resonance images of the thoracolumbar vertebral column in a 6-year-old male neutered cat at the level of T2 (A) and L2 vertebrae(B). The epaxial muscles in both locations contain patchy contrast enhancement (red arrows).

One week after discontinuation of clindamycin, the cat started drooling profusely and had difficulty closing the mouth when prehending food. After restarting clindamycin these signs improved, but 5 days later, the cat developed right pelvic limb (RPL) paresis with proprioceptive deficits, decreased withdrawal and patellar reflexes. Lumbar spinal pain suggested a lesion affecting the lumbar intumescence or right lumbosacral plexus. Mild right medial iliac and colonic lymphadenomegaly was visualized on abdominal ultrasound. MRI of the lumbosacral spine revealed diffuse contrast enhancement of several muscles. Lumbar CSF albuminocytological dissociation (NCC: 1/uL, protein concentration: 0.65 g/L) persisted. Electromyographic and nerve conduction velocity (NCV) study (Nicolet Viking Quest, MFI Medical, San Diego, CA, USA) abnormalities revealed changes consistent with a myopathy and neuropathy (Supplemental documents). Right cranial tibial and triceps muscle biopsies, evaluated in frozen and paraffin fixed sections, showed a mild to moderate multifocal myositis with several myofibers containing large parasite cysts measuring 150–200 µM in diameter consistent with *Sarcocystis* sp. (Figure 3). Mononuclear cell infiltrations were at sites distant from the parasite cysts and not directly invasive. A pan-fungal blood PCR panel and CSF *Sarcocystis neurona* titers were negative. Immunohistochemistry and PCR for *Bartonella* spp. on formalin fixed and fresh frozen muscle samples were negative. A PCR targeting the 18S ribosomal RNA gene was performed on DNA extracted from frozen muscle tissue (methods in Supplemental documents). National Center for Biotechnology Information (NCBI) Basic Local Alignment Tool nucleotide (BLASTn) analysis of the 18S rRNA gene was 99.9% identical (744/745 bp) to *Sarcocystis caninum* (Gen Bank accession# MH469240.1) and *Sarcocystis artica* (Gen Bank accession# MH469240.1), and 99% identical (339/340 bp) to *Sarcocystis felis*. A cox1 amplicon was 99.4% identical (321/323 bp) to *S. caninum* (Gen Bank accession# MH469240.1) and to *S. artica* (Gen Bank accession# MF596306.1). A Gen Bank cox1 reference sequence was not available for alignment with *S. felis*. Other cox1 *Sarcocystis spp*. with 99.7% identity (322/323 bp) included: *Sarcocystis fulicae* (Gen Bank accession# MH138316.1), *Sarcocystis wobeseri* (Gen Bank accession# MH138315.1), *Sarcocystis corvusi* (Gen Bank accession# MH138314.1), and *Sarcocystis cornixi* (Gen Bank accession# MH138313.1). Due to poly-repetitive regions, only a smaller ITS-1 sequence was available in Gen Bank for BLAST analysis (525 bp). When aligned with *S. felis* (Genbank accession # AY190081.1 and AY190082.1) cloned ITS-1 amplicons varied between 97.3% and 99.0% (Table 1). Based upon cumulative DNA sequence alignments of multiple gene targets we suspect that the visualized organism was *S. felis,* but infection with another Sarcocystis sp. more similar to *S. caninum* could not be ruled out. Treatment was directed at a co-infection with *B. henselae* and *S. felis*.

**Figure 3.**
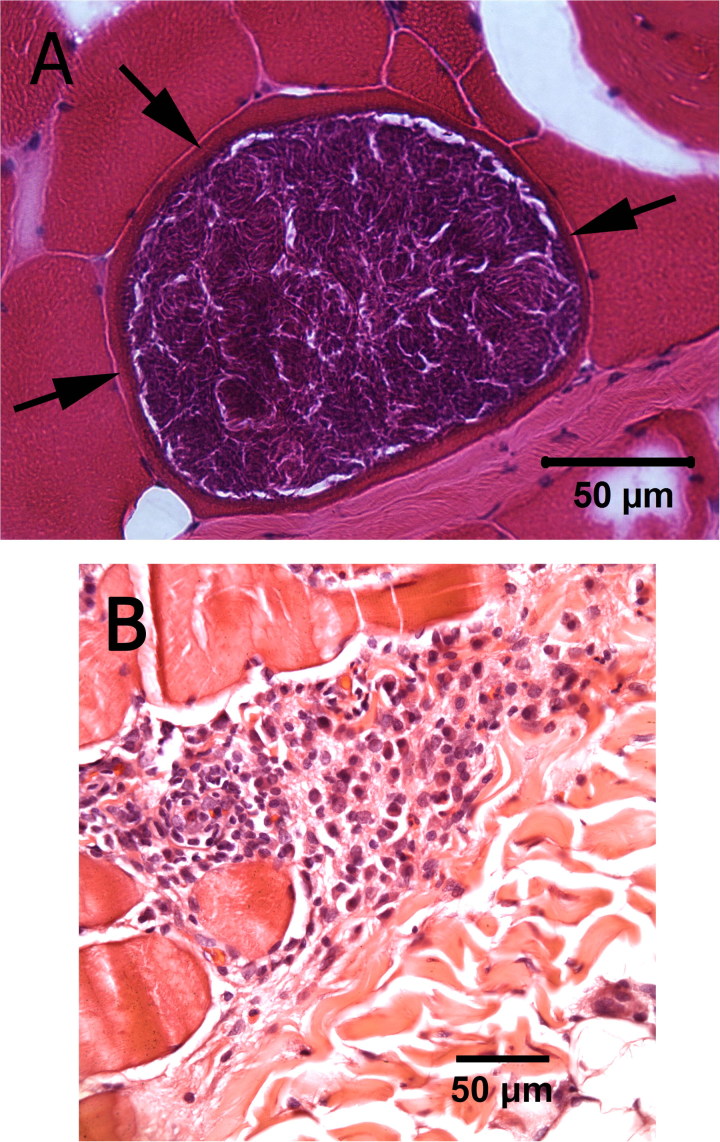
High power image of a Sarcocystis cyst in a muscle fiber from a cryosection of the right cranial tibial muscle in a 6-year-old male neutered cat (A). Arrows indicate margins of the muscle fiber containing the cyst. Mononuclear cell infiltrates did not directly invade the infected muscle fiber but were present in areas distal to the cysts (B), (H&E stain).

**Table 1.** Poly-repetitive ITS-1 region sequences amplified from DNA of biopsy of the muscle tissue from a 6-year-old male neutered cat. Sequence homologies calculated against all known Sarcocystis felis ITS-1 Genbank accessions to date.

		*Sarcocystis felis* ITS-1 homology	
		GenBank Accession	
Sample ID (ITS-1 clones)	GenBank Accession	AY190081.1	AY190082.1
SarcoR4115-clone1	MN508375	518/525 bp (98.7%)	513/525 bp (97.7%)
SarcoR4115-clone2	MN508376	511/525 bp (97.3%)	520/525 bp (99.0%)
SarcoR4115-clone3	MN508377	515/525 bp (98.1%)	514/525 bp (97.9%)
SarcoR4115-clone4	MN508378	512/525 bp (97.5%)	520/525 bp (99.0%)
SarcoR4115-clone5	MN508379	517/525 bp (98.1%)	514/525 bp (97.9%)

The cat was treated with Trimethoprim Sulfadiazine (TMS) (24 mg/kg BW every 12 h PO). Meloxicam (Metacam, [Boehringer Ingelheim, St. Joseph, MO, USA] 0.1 mg/kg BW every 24 h PO) and buprenorphine (0.015 mg/kg BW every 8 h sub-lingual) were prescribed for analgesia for 5 and 3 days, respectively following muscle biopsies. Five days later, the owner reported some improvement but the cat was still knuckling on its RPL. Ten days later, there was a transient relapse characterized by dysphagia, recurrence of anisocoria and ataxia. Pradofloxacin (5 mg/kg BW every 24 h PO) was added for treatment of the *B. henselae* infection. One week later, the cat developed paresis in three limbs. Because of difficulties with administration of liquid medication, ciprofloxacin (25 mg/kg BW every 24 h PO) was substituted for pradofloxacin after 4 days of treatment. After 10 days, the paresis worsened so pyrimethamine (2 mg/kg BW every 24 h PO) was added. Five days later, the cat became non-ambulatory tetraparetic, lethargic, painful and anorexic. Gabapentin (10 mg/kg BW every 12 h PO) was added for pain management. Due to anorexia, the ciprofloxacin was discontinued and appetite returned. Three days later, the cat started to improve neurologically and was ambulatory tetraparetic with weak motor function in the RTL and strong motor function in the other limbs. Proprioceptive deficits in all limbs and mild anisocoria persisted. Two weeks later, neurological abnormalities continued to improve, but the cat had developed a non-regenerative anemia, neutropenia, and moderate increase in alanine aminotransferase (ALT) and aspartate aminotransferase (AST) activities (Supplemental documents). These changes resolved 4 weeks after discontinuation of TMS and Pyrimethamine. Three months later, neurological examination, CBC and serum chemistry results were normal, with the exception of mild hyperglobulinemia. Titers for *B. henselae* were positive, but a BAPGM enrichment blood culture was negative. A comprehensive tick serology and PCR panel (NCSU Vector Borne Disease Feline Comprehensive Panel) to rule out untreated concurrent infections was negative. This panel includes serology testing for *B. vinsonii* subsp. *berkhoffii, Bartonella koehlerae* (by IFA) *Borrelia burgdorferi*, *Anaplasma,* and *Ehrlichia* spp. antibodies and *Dirofliaria immitis* antigen (Snap^®^4DX Plus^®^ IDEXX Laboratories, city, state, USA) paired with PCR testing to detect the presence of *Anaplasma, Babesia, Bartonella, Cytauxzoon, Ehrlichia, Rickettsia*, and hemotropic *Mycoplasma* organism DNA. *Hepatozoon* spp. and *S. neurona* PCR on blood extracted DNA were negative. *Neospora caninum* PCR on muscle was also negative. The cat remained neurologically normal with symmetrical pupils two years later with only residual signs of mild dysphagia.

This unique case documents co-infection with *B. henselae* and one or two *Sarcocystis sp.* in an adult cat with multifocal neuromuscular deficits, spastic pupil syndrome and chorioretinitis. The contributions of each agent, versus the influence of co-infection on the pathogenesis of the neuromuscular and ocular lesions in this cat were impossible to determine in the clinical setting. Treatment using fluoroquinolones, TMS and pyrimethamine accompanied resolution of the neurologic signs.

Cats are considered intermediate hosts in the life cycle of *S. felis* (Dubey et al. 2000). That organism has been attributed a questionable pathogenic role and only following immunosuppression (Dubey et al. 1994; Gillis et al. 2003; Greiner et al. 1989). In our case, the muscle protozoal cysts were further characterized by PCR. We generated DNA sequences for three target genes suggesting that the muscles contained organisms that were closely related to *S. felis,* but also to *S. caninum* and *S. artica*. PCR amplification, cloning and DNA sequencing supported *B. henselae* and *Sarcocystis* co-infection. To date, *S. felis* had not been associated with disease in cats (Little 2014), therefore, our findings potentially support co-infection with more than one *Sarcocystis* sp. Given the higher prevalence of *S. caninum* in our region, an infection with this *Sarcocytis* sp., as well as *S. felis,* was considered more likely than *S. arctica*. However, *S. arctica* infection could not be completely excluded, as these *Sarcocystis* organisms are very closely related on an evolutionary basis. When aligned with available Gen Bank nucleic acid sequences, differences among the targeted genes suggested the presence of more than one *Sarcocystis* spp. PCR testing did not support infection with another protozoal genus and DNA sequences were not consistent with *S. neurona,* a species associated with muscular disease.

Sarcocystis infections causing encephalomyelitis have been previously reported in three kittens (Dubey et al. 1994; Bisby et al. 2010; Dubey, Benson, and Larson 2003). In one of these cases, *Sarcocystis neurona* was diagnosed on the basis of PCR and DNA sequence analysis and initial treatment with clindamycin also failed to improve the kitten’s ataxia, spinal pain and anisocoria, whereas the addition of pyrimethamine and TMS accompanied resolution of neurological and ocular signs (Bisby et al. 2010). Similar to our adult cat, the kitten developed leukopenia, increased ALT and AST activities suspected to be related to the antibiotics, as discontinuation after 20 days of treatment was followed by normalization of the hematologic and serum chemistry abnormalities. The increased ALT and AST activities could have been secondary to muscle necrosis as reported with Toxoplasma infection (Dubey, Lindsay, and Lappin 2009). However, serum creatine kinase (CK) activity was not elevated at any time point (Supplemental documents). Our cat developed a non-regenerative anemia suspected to be secondary to the pyrimethamine. Folinic acid supplementation might have prevented the anemia, but difficulties in administering oral medications precluded administration.

Despite normal CK values, the multifocal myositis identified in this cat was evident on MRI and subsequently confirmed by histopathological examination of muscle biopsies. Although MRI changes associated with central nervous system (CNS) toxoplasmosis have been reported (Pfohl and Dewey 2005; Falzone et al. 2008; Alves et al. 2011), MRI changes in association with an infectious myositis have not been described in cats yet to our knowledge. Identification of similar changes in future cases warrants muscle biopsies and thorough infectious disease testing. The electrodiagnostic changes confirmed concurrent peripheral nerve as well as muscle involvement. Elevated CSF protein concentrations suggested CNS involvement, although we cannot say if this change was due to *Sarcocystis* infection, neurobartonellosis or co-infection.

Because domestic cats are considered the primary reservoir for *B. henselae* and are usually asymptomatic, the true contribution of this bacteria to development of chronic illness remains unclear (Guptill 2010; Guptill et al. 1999; Kordick et al. 1999). *Bartonella henselae* has been associated with long standing intravascular infection in cats potentially resulting in chronic inflammatory diseases (Guptill 2010; Breitschwerdt et al. 2010). There is also increasing support for variation in virulence among *B. henselae* strains infecting cats and humans (Breitschwerdt 2017). As mentioned before, *Sarcocystis* sp. clinical infection in cats has been associated with immunosuppression (Dubey et al. 1994; Greiner et al. 1989; Dubey, Benson, and Larson 2003). In order to explain the chronic waxing and waning signs in this cat, we hypothetized that relapsing *B. henselae* bacteriemia may have decreased the cat’s immune competence and caused a normally quiescent protozoal infection to induce intermittent clinical signs (Kabeya et al. 2009).

As is common with chronic infections, we could not determine when and how long each infection had persisted in this cat. The owner reported that the cat had suffered from anisocoria since a kitten and based upon the repeated documentation of hyperglobulinemia and eosinophilia, it is likely that one or both infections were present for at least a year. Eosinophilia occurred in cats experimentally-infected with *B. henselae* (Kordick and Breitschwerdt 1997) and hyperglobulinemia has been associated with *B. henselae* seroreactivity in naturally-infected cats (Whittemore et al. 2012). The hypocholesterolemia was thought to be a consequence of down-regulation of cholesterol production by the liver in the face of increased globulin production to maintain oncotic pressure (Patel et al. 2005). Assuming no in-house exposures, it is possible that the cat was *B. henselae* bacteremic and hosted a quiescent protozoal infection since rescued as a stray kitten. Clearly, concurrent infections contribute to complex disease expression, in association with overlapping clinical, hematological and biochemical abnormalities (Maggi et al. 2013).

The changing anisocoria in this case was suspected to be a consequence of spastic pupil syndrome or, less likely, secondary to involvement of the sympathetic innervation of the eyes from either myelitis affecting the preganglionic neuronal cell body in the high thoracic spinal cord or inflammation of the vagosympathetic trunk. Spastic pupil syndrome, has been reported in cats positive for FeLV but our case tested negative multiple times (Cullen and Aubrey 2013b). It is unlikely for the anisocoria to have been secondary to chorioretinitis as the miosis would be abolished in the dark and pupillary light reflexes would be abnormal with only retinal lesions (Cullen and Aubrey 2013a). It is unclear which, if either, of the infectious agents caused the ocular signs. However, the fact that the cat’s anisocoria had been present for 6 years and resolved after treatment indirectly supports a role of chronic infection in its pathophysiology.

The cat worsened following initiation of treatment. In dogs treated for *Bartonella* species, a Jarisch Herxheimer-like reaction suspected to be secondary to bacterial death causing massive cytokine release and characterized by lethargy, and signs resembling sepsis is reported about 4-7 days after starting antibiotics (Diniz 2014). A similar phenomenon may occur following destruction of intramuscular protozoal cysts and explain the initial worsening of signs in our case. Administration of anti-inflammatory drugs might be beneficial to prevent this reaction and avoid early treatment discontinuation. The cat was treated with a fluoroquinolone for 19 days, during which TMS was concurrently administered. Because *Bartonella* bacteremia can have a relapsing pattern, we cannot exclude the possibility that the cat remained infected despite the negative post-treatment blood culture (Kordick et al. 1999; Breitschwerdt et al. 2010; Regnery et al. 1996). Fortunately, the cat made a full recovery despite earlier antibiotic discontinuation than recommended (Breitschwerdt et al. 2010), suggesting that the duration of treatment and/or the antibiotic combination was adequate to address both infections.

Upon detailed examination of the muscle biopsies, we could not find any evidence of free tachyzoites nor did we see any proliferation of the sarcosporidia. However, we believe, based on the response to the TMS and pyremithamine, that there must have been active cysts with ongoing proliferation of *Sarcocystis sp.* organism as these two drugs have no effect on bradyzoites.

One of the limitations of this case is that we did not run any serology for *Neospora caninum* or *Hepatozoon sp*. However, PCR on blood for *Hepatozoon* was negative and PCR on muscle tissue for *Neospora* was also negative.

In conclusion, veterinarians should include *Sarcocystis sp*. and *B. henselae* as differential diagnostic considerations for diffuse neurological signs, myositis, spastic pupil syndrome and chorioretinitis. Treatment directed at both organisms is recommended and an initial worsening of clinical signs followed by improvement and disease resolution is possible.

## Supplementary Material

Supplemental Material
