# Supplementary material for: Co-infection with Bartonella henselae and Sarcocystis sp. in a 6-year-old male neutered domestic longhair cat with progressive multifocal neurological signs
Source: Vet Q. 2019 Dec 10;39(1):168–73. doi: 10.1080/01652176.2019.1697012 (PMC6913637; doi:10.1080/01652176.2019.1697012)
Supplement: Supplemental Material [file TVEQ_A_1697012_SM7385.docx]

Table 2. Major hematological and serum biochemical abnormalities documented in a 6-year-old male neutered domestic long haired cat. “NA” indicates results not available. Abnormal results are highlighted in bold.

|  | Reference interval | 02/14 | 04/14 | 02/15 | 03/15 | 04/15 | 05/15 | 08/15 |  |
| --- | --- | --- | --- | --- | --- | --- | --- | --- | --- |
| Neutrophils  (/uL) | 2773-6975 | NA | **10582** | 7712 | 7697 | **12609** | **1509** | 7995 | |
| Eosinophils  (/uL) | 118-879 | **2546** | **2.605** | **2072** | **2919** | **1555** | 315 | 513 | |
| Lymphocytes  (/uL) | 415-4996 | NA | 1954 | 1266 | 1327 | 755 | 1044 | 1333 | |
| Basophils  (/uL) | 0-355 | NA | 0 | 115 | **398** | 0 | 0 | 0 | |
| RBC  (/uL) | 7120-11460 | NA | 8030 | 7430 | 8030 | 7990 | **5880** | 7410 | |
| Globulin  (g/L) | 0.29-0.48 | **0.62** | **0.69** | **0.74** | **0.68** | NA | 0.4 | **0.53** | |
| Cholesterol  (g/L)  (mmol/L) | 8.9-28.7  2.3-7.4 | **6**  **1.5** | **8.5**  **2.1** | **6.7**  **1.7** | **6.9**  **1.8** | NA | 9  2.34 | 8.9  2.3 | |
| ALT  (IU/L) | 27-158 | NA | NA | 34 | 33 | NA | **1423** | 29 | |
| AST  (IU/L) | 16-67 | NA |  | 18 | NE | NA | **696** | NE | |
| CK  (IU/L) | 60-531 | NA |  | 260 | 291 | NA | 163 | 142 | |

Figure 4**:** Picture of the right (A) and left (B) fundus showing hyperreflective lesions of the tapetum consistent with chorioretinitis.


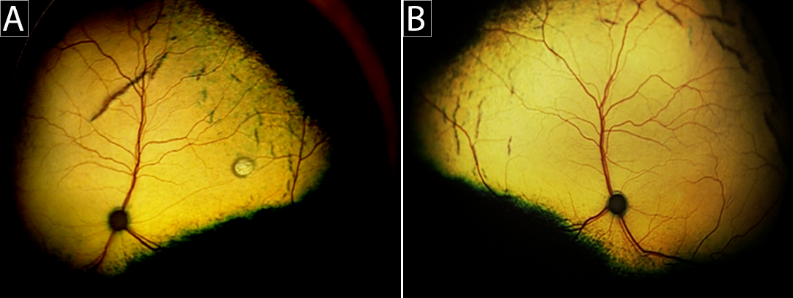


Figure 5: Electrodiagnostic findings.


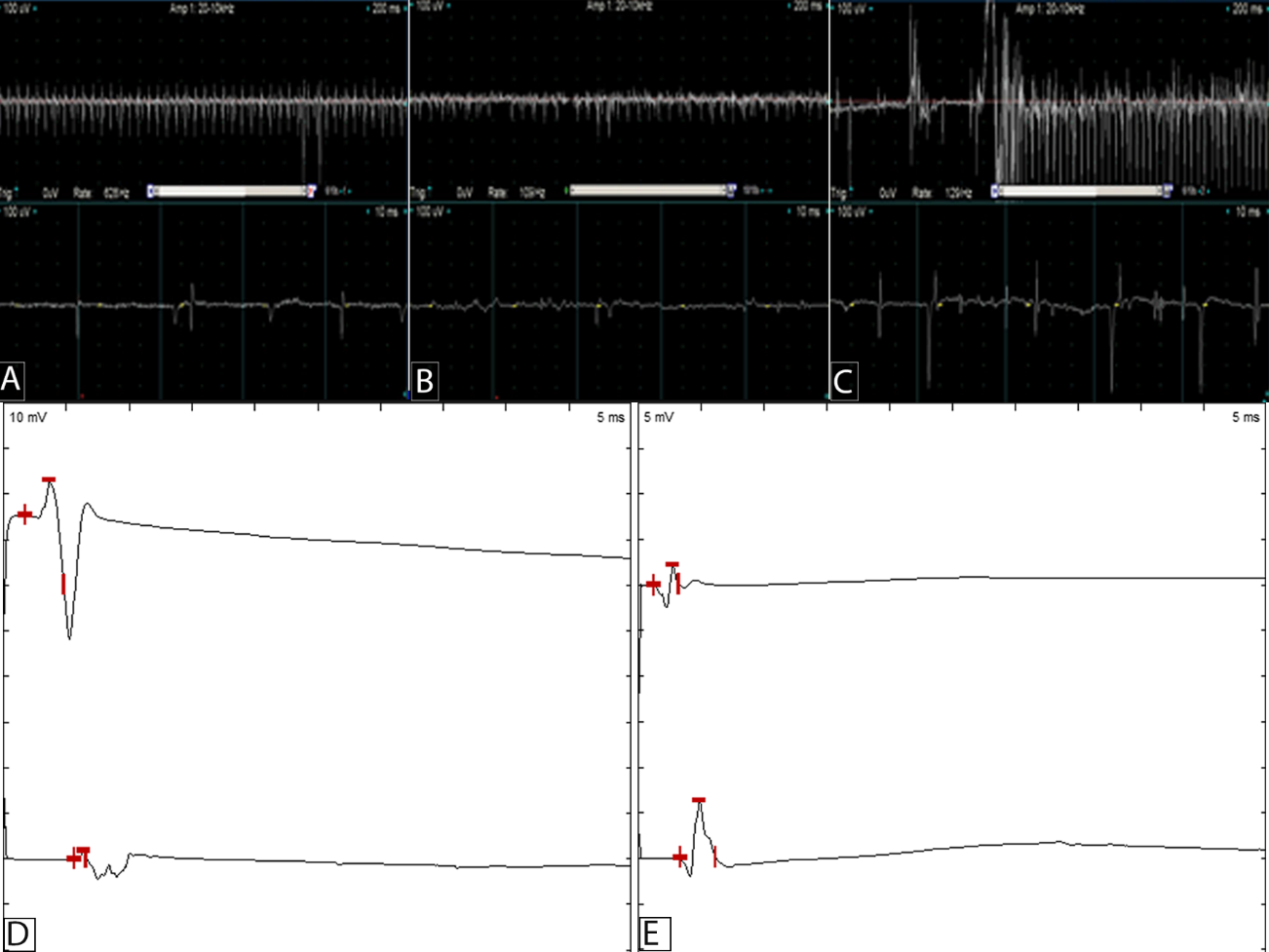
Electromyography documented spontaneous activity (fibrillation potentials and positive sharp waves) in the right palmar interosseous (A), left palmar interosseous (B), left extensor digitalis, left biceps brachii, left extensor carpi radialis (C) and left supraspinatous muscles. The motor nerve conduction study identified decreased amplitude (7.6mV distally and 1.8mV proximally, reference 20.9 and 15.6 respectively(25)) and decreased distal conduction velocity (64m/s, reference interval 86.4-133.7m/s(25)) without temporal dispersion measured from the right plantar interosseous muscle following sciatic/tibial nerve supramaximal stimulation (D). Similar findings were noted following left ulnar nerve stimulation with a decreased amplitude (distal 2.2mV, proximal 6.3 mV, reference 18.8 and 15.7 respectively(25)) and nerve conduction velocity (50m/s, reference interval 60.2-124.2(25)) (E).

**Methods for *Sarcocystis* PCRS**

A PCR targeting the *18S ribosomal RNA* gene was performed on DNA extracted from frozen muscle tissue. To further delineate the *Sarcocystis* sp., mitochondrial DNA was tested using a quantitative (qPCR) assay designed to amplify a 379 bp region of the cytochrome c oxidase subunit I (cox1) gene using forward primer (Sarco_cox1_53F 5’-TAT ATT TAG TGT WGT TGG TAC TCT GAT GTC-3’) and reverse Sarco_cox1_432R 5’-GAT CCA GTC TAC GGA TTC CGT ATT CA-3’) and a 879 bp region of internal transcribed spacer *(*ITS-1) region (Kutkiene et al.,2010). All PCRs included water as a non-template negative control and a known-positive *Sarcocystis* DNA sample as a positive control. Amplification was performed in CFX96™ Real-Time Detection System combined with C1000™ Thermal Cycler (Bio-Rad, USA) for qPCR and an Eppendorf Mastercycler EPgradient® with aluminum block for conventional PCR (cPCR). Amplification reactions contained 12.5 µL SYBR^®^Green Supermix (Bio-Rad, Hercules, CA, USA) for qPCRs or MyTaq HS Mix (2X) (Bioline, London, U.K. cat: BIO-25046) for cPCRs. Thermocycler conditions consisted of an initial denaturation step at 98 °C for 3 min, followed by 40 cycles of (i) 98 °C for 15 s, (ii) annealing temperature at 57 °C for 15 s, and (iii) 72 °C for 20 s. Melting temperature (T_m_) measurements were made between 65–95 °C at 0.5 s intervals for qPCRs. Sequencing was performed by GENEWIZ, Inc. (Raleigh, NC), and AlignX software (Vector NTI Suite 6.0, InforMax, Inc., Bethesda, MD, U.S.A.) was used to align amplicon DNA sequences with reference sequences from GenBank.

**Reference:**

# Kutkiene L, Prakas P, Sruoga A, Butkauskas D. The mallard duck (Anas platyrhynchos) as intermediate host for Sarcocystis wobeseri sp. nov. from the barnacle goose (Branta leucopsis). Parasitol Res. 2010;107:879-88.
